# Evaluation of *Brachypodium* spp. System Model Against *Fusarium poae*

**DOI:** 10.3390/jof11010032

**Published:** 2025-01-04

**Authors:** Florencia Arroyo, Mauro Martínez, Agustín Arata, María V. Moreno, Marie Dufresne, Sebastián A. Stenglein, María I. Dinolfo

**Affiliations:** 1 Instituto de Biología Funcional y Biotecnología (BIOLAB)-INBIOTEC-CONICET-CICBA, Facultad de Agronomía, Universidad Nacional del Centro de la Provincia de Buenos Aires, Av. República de Italia # 780, Azul 7300, BA, Argentina; arroyoflor88@gmail.com (F.A.); maurom@azul.faa.unicen.edu.ar (M.M.); arataa@faa.unicen.edu.ar (A.A.); vmoreno@azul.faa.unicen.edu.ar (M.V.M.); 2 Mejoramiento Genético Vegetal, Facultad de Agronomía de Azul, UNCPBA, Azul 7300, BA, Argentina; 3 Área de Cereales y Oleaginosas, Facultad de Agronomía de Azul, UNCPBA, Azul 7300, BA, Argentina; 4 INRAE, UR BIOGER, Université Paris-Saclay, 91120 Palaiseau, France; marie.dufresne@universite-paris-saclay.fr

**Keywords:** plant–pathogen interactions, *Fusarium poae*, disease severity, *Brachypodium* spp.

## Abstract

Cereal crops are affected by one of the most devastating diseases worldwide, known as Fusarium head blight (FHB), with *Fusarium graminearum* being the most isolated causal pathogen. Another species associated with this disease is *Fusarium poae*. This species has been considered a relatively weak pathogen compared to *F. graminearum*, but its importance has increased due to its occurrence in cereal grains worldwide. Considering the advantages of using *B. distachyon* as a plant model and the importance of *F. poae* in crops, our study aimed to evaluate the potential use of *Brachypodium* as a plant model to evaluate the compatible interaction with *F. poae*. Twelve *Brachypodium* spp. accessions from different countries were inoculated with a selected *F. poae* set of isolates. Disease severity, conidial quantification, fungal DNA biomass, and nivalenol quantification were assessed. The results showed a compatible interaction between *Brachypodium* accessions and *Fusarium poae*, which allowed the use of the model plant for future plant–pathogen interaction studies.

## 1. Introduction

In the past several years, shifting climates have increased the number of diseases caused by pathogens, which are the major contributors to worldwide health insecurity [1]. Crops with agronomically relevant traits, such as barley, wheat, oats, and maize, are affected by multiple phytopathogenic fungi [2,3,4,5]. One of the most harmful fungi in crops globally belongs to the genus *Fusarium* [6]. These species of fungi are globally distributed but are prevalent in particular localities depending on the climate patterns and cropping systems [7]. In Argentina, wheat and barley are the main large-area winter crops and are affected by one of the most devastating diseases worldwide, known as Fusarium head blight (FHB) [8,9]. Globally, among the most relevant species of the *Fusarium* genus, those belonging to the *Fusarium sambucinum* species complex (FSSC) are the most frequent in small grain cereals [10]. At the molecular level, this complex consists of at least 35 species, but *Fusarium graminearum* is the main disease responsible worldwide [11,12,13]. *Fusarium* spp. can infect several parts of the plants in different developmental stages [14]. Moreover, *Fusarium graminearum* is also responsible for other diseases such as Fusarium root rot (FRR) and seedling blight [15,16]. Other *Fusarium* species associated with FHB worldwide are *F. cerealis* (syn. *F. crookwellense*), *F. culmorum*, *F. poae*, and *F. pseudograminearum* [17,18,19]. In wheat, symptoms are associated with the heads (ears) of the infected plants which develop bleaching spikes and often do not produce grains or, in some cases, produce shrivelled kernels with light weight [20,21].

At present, *F. poae* isolates have become relevant for their occurrence in cereal grains [22]. This fungus has been considered a relatively weak pathogen compared to *F. graminearum* [23,24]. *F. poae* affects glumes or grains, but interest has increased because of the lack of visible symptoms on spikelets after infection [25]. Most recent studies show that this fungus is frequently isolated from barley grain in different parts of the world, with a high incidence in Argentina [25,26]. In addition, this pathogen can produce a large number of mycotoxins potentially harmful to the health of humans and animals [27]. These secondary metabolites are present in food and feed prepared from contaminated cereal grains [28]. Nivalenol (NIV) is one of the most important mycotoxins belonging to type B trichothecenes that can be produced by *F. poae*, making this pathogen even more relevant [29]. Other mycotoxins are type A trichothecenes, such as diacetoxiscirpenol (DAS) and neosolaniol (NEO) [21]. Moreover, several works have reported the ability of *F. poae* to produce toxin T-2 and toxin HT-2. However, it was recently demonstrated by Witte et al. [24] that the *TRI16* gene responsible for T-2 and HT-2 production is not intact in *F. poae* isolates; therefore, they concluded that this species cannot produce these harmful mycotoxins.

Plant–pathogen interaction studies are crucial to understanding how the plant pathogen accesses the resources necessary for its growth, reproduction, and survival within the host [30]. Therefore, hosts have developed different mechanisms to control their resistance/susceptibility to infection for pathogens [31]. In this field of research, cereal crops pose difficulty in studying interactions because of their large complex genomes [32]. Furthermore, the size of these crops makes it difficult to work in laboratories [33]. Therefore, *Brachypodium distachyon* (L. P. Beauv.) has been proposed as a model pathosystem for research on cereal crop diseases [34]. Genetic surveys have revealed that *B. distachyon* is more phylogenetically related to wheat and barley than other grasses such as rice or maize. Genomically, this synteny allows the transfer of *Brachypodium* information to *Triticaceae* genotypes [35]. *B. distachyon* is a monocot member of the *Poaceae* (Gramineae) family with characteristics of biological interest, including a small genome (approximately 271 Mbp), self-fertility, small stature, and a short generation time [36]. In the past decade, *B. distachyon* has emerged as a model plant for its capacity to host many cereal pathogens and develop symptoms of infection such as FHB [37,38]. It is known as the sequenced genome of accession Bd 21, a line sequence used as a reference [39,40]. Several researchers have shown these plant physiological and genetic advantages as a comparative and functional genetic model for agricultural studies [3,15,32,34].

Therefore, considering the advantages of using *B. distachyon* as a plant model and the importance of *F. poae* in crops, the present study aimed to evaluate the potential use of *Brachypodium* as a plant model to evaluate its compatible interaction with *F. poae*.

## 2. Materials and Methods

### 2.1. Fusarium poae Isolates Selection

**Figure 1:**
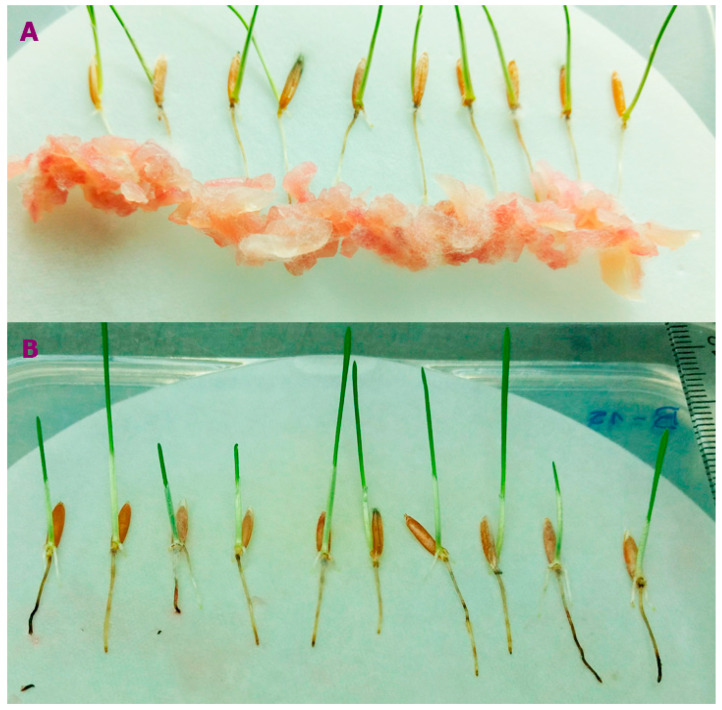
Selection of *F. poae* isolation based on the susceptibility of *Brachypodium* spp. roots. (**A**) Slurry of *Fusarium poae* on Pakistan accession roots to measure the necrotic area. (**B**) *B. distachyon* inoculated with *F. poae* showing necrosis symptoms.

A total of eight isolates of *F. poae* were obtained from the fungal collection of the Instituto de Biología Funcional y Biotecnología (BIOLAB-Azul, Buenos Aires province, Argentina). These fungal isolates were first identified macro- and microscopically [41] and were molecularly confirmed by using PCR species-specific primers [42]. Four *F. poae* isolates from barley grains and were identified as 15-22.2-25.5-46.2 [26], and the other four isolates originated from wheat grains identified as 37 (TSa1b)-40 (TBig1a)-43 (TMa1a)-47 (TPe1a) [28,43]. Two accessions of *Brachypodium* were selected based on the susceptibility of roots to different *Fusarium* species, showing that the Bd-21 and Pakistan accessions have greater tolerance and susceptibility to *F. poae*, respectively [3]. Seeds were pre-germinated (ten seeds per accession) in square plastic Petri dishes containing filter paper on a damp surface of 0.8% water agar for approximately five days at 4 °C. Subsequently, they were incubated vertically for 10 days at 22 °C under a photoperiod of 16 h light/8 h arkness. A total of 30 roots with a similar length (three replicates per isolate per *Brachypodium* accession) under sterile conditions were inoculated. Inoculum was prepared using the protocol of Goddard et al. [44]. The *Fusarium* slurry was deposited 1 cm below the roots using a small sterile syringe (0.1 a 5 mL) (Figure 1A). At two days post-inoculation (dpi), seedling roots were washed with sterile distilled water to remove the inoculum residues. Roots were photographed at 2, 4, 6, and 8 dpi (Figure 1B) to measure necrosis using ImageJ software (ImageJ 1.54f) [45]. The area under the disease curve (AUDPC) was calculated using the trapezoidal integration method [46], and the necrosis area on the root was expressed as a percentage of the necrosis area under the total root area. ANOVA analyses were performed, and the significance levels were established using Tukey test at *p* < 0.05.

### 2.2. Brachypodium Accessions

The *Brachypodium* accessions used came from different parts of the world: tree diploid *B. distachyon* from United States of America (USA) (WA 36678), Turkey Adi-7 (WA 39240), and Bd-21 from Iraq (PI 254867), and nine hexaploid *B. hybridum* from Afghanistan (PI 219968), Australia (PI 533015), Iran (PI 239714), Iraq (PI 254868), Israel (PI 233228), Pakistan (PI 250647), South Africa (PI 208216), Spain (PI 287783), and Uruguay (PI 372187). For each trial, seeds of each *Brachypodium* accession were disinfected with sodium hypochlorite (0.6% *v*/*v*) for ten minutes with continuous shaking and three washes with sterile distilled water for ten minutes.

### 2.3. Pathogenicity Assay and F. poae Inoculation

A total of 5 seeds per 5 L pots filled with clay loam soil were sowed, irrigated, and drained to maintain humidity under greenhouse conditions where the minimum and maximum temperatures were registered daily. For inoculation, the selected *F. poae* isolate was cultured in Petri dishes containing 2% PDA and was incubated at 25 °C ± 2 °C with 12 h light/12 h darkness. For the conidial harvest, 5 mL of distilled water was added on the PDA medium, and the conidia were taken with a bent glass rod. The suspension was filtered using cheesecloth, and the conidial suspension was adjusted to 1 × 10^5^ conidia per mL [47], using a haemacytometer (Biopack, Buenos Aires, Argentina) and a binocular microscope. Tween^®^ 20 (0.05%) (Biopack) was added as a surfactant. Floral point inoculation was made when spikelets reached anthesis (around 30–35 days after sowing). For this purpose, 3 µL of *F. poae* inoculum was applied in the central part of the floral cavity in the second spikelet starting from below. The control treatment was applied using water plus Tween^®^ 20 (0.05%) (Biopack). After inoculation, plants were covered with polypropylene bags for 2 days to improve fungal growth and humidity conditions. The design was completely randomized with eight replicates.

### 2.4. Disease Severity and Quantification of Fungal Sporulation

Disease severity (DS) was evaluated at 7, 14, and 21 days post-inoculation (dpi) considering the following symptom score: 0 (no visible symptom), 1 (presence of symptom in the inoculated cavity of the spikelet), 2 (presence of symptom not only in the inoculated cavity but also in 1 or 2 neighboring cavities of the spikelet), 3 (presence of symptom in 3 or more cavities continuous to the inoculated one), and 4 (the whole spikelet with symptoms and symptoms in adjacent spikelets) [48].

For conidial quantification, spikes were collected (five spikes per accession) at 7, 10, 14, and 21 days post-inoculation (“dpi”) and were transferred in sterile Erlenmeyers containing 15 mL of sterile distilled water. Spikes were vigorously shaken for 30 min at 180 rpm in sterile distilled water to resuspend conidia. Finally, the obtaining suspension was filtered, and conidia were quantified (conidia/mL) using a Neubauer haemacytometer and a binocular microscope (Olympus CX 31^®^, Miami, FL, USA).

For DS and conidial quantification, ANOVA analyses were performed, and the significance levels were established using Tukey test [49].

### 2.5. Fungal Genomic DNA Quantification

A total of 5 spikes with three replicates were collected at 21 dpi and placed in liquid nitrogen until DNA extraction [50]. The DNA quantity was calculated using a fluorometer (Qubit Fluorometer, Invitrogen, Buenos Aires, Argentina). A standard curve with pure *F. poae* DNA was built with known concentrations of 10 ng/µL to 0.001 ng/µL. Quantification of *F. poae* DNA was made by qPCR on 10 ng of total DNA using *F. poae* specific primers: FpoaeA51 fwd (5′-ACC GAA TCT CAA CTC CGC TTT-3′) and FpoaeA98 rev (5′-GTC TGT CAA GCA TGT TAG CAC AAG T-3′) [51]. PCR reactions were performed twice on an Applied Biosystems 7500 real-time PCR system (Thermo Fisher Scientific, Waltham, MA, USA) using the following cycling protocol: 2 min at 50 °C; 95 °C for 10 min; 40 cycles of 95 °C for 15 s; and 60 °C for 1 min followed by dissociation curve analysis at 60 to 95 °C. SsoAdvancedTM Universal SYBR^®^ Green Supermix (BIO-RAD, Hercules, CA, USA) was used to reveal amplifications.

### 2.6. NIV Quantification

*Brachypodium* spikes at BBCH 97 were taken for NIV quantification and finely ground in a laboratory grinder (Arcano Fw-100 high-speed universal disintegrator) [52]. A volume of 20 mL of extraction solvent (CH3CN/H2O/Hac 79 + 20 + 1) was added to 1 g of ground *Brachypodium* spp. spikes. The samples were homogenized with the Ultraturrax for 3 min, sonicated for 60 min, and finally centrifuged for 5 min at 3000 rpm. A volume of 10 mL of extract was transferred into glass vials and evaporated to dryness at 45 °C under a stream of N2. Samples were resuspended in methanol/water (70:30) and filtered through a 0.22 mm nylon filter before analysis. Nivalenol was identified and quantified using high-performance liquid chromatography coupled with tandem mass spectrometry (HPLC MS/MS) with a detection limit of 10 ng/g and a quantification limit of 30 ng/g [53]. The analyses were conducted in a Thermo Scientific™ system consisting of a degasser, quaternary pump, column oven, and an LTQ XL™ ion trap mass spectrometer (Thermo Fisher Scientific, Waltham, MA, USA). Chromatographic separations were performed with a C18 100 × 2.1 mm Hypersil™ ODS (5 mm particle size) column. A solution of ammonium formate in acetonitrile (10 mM) was used as a mobile phase. Samples (10 mL) were analyzed at a 0.2 mL/min flow rate at 45 °C.

## 3. Results

### 3.1. Fusarium poae Selection

**Figure 2:**
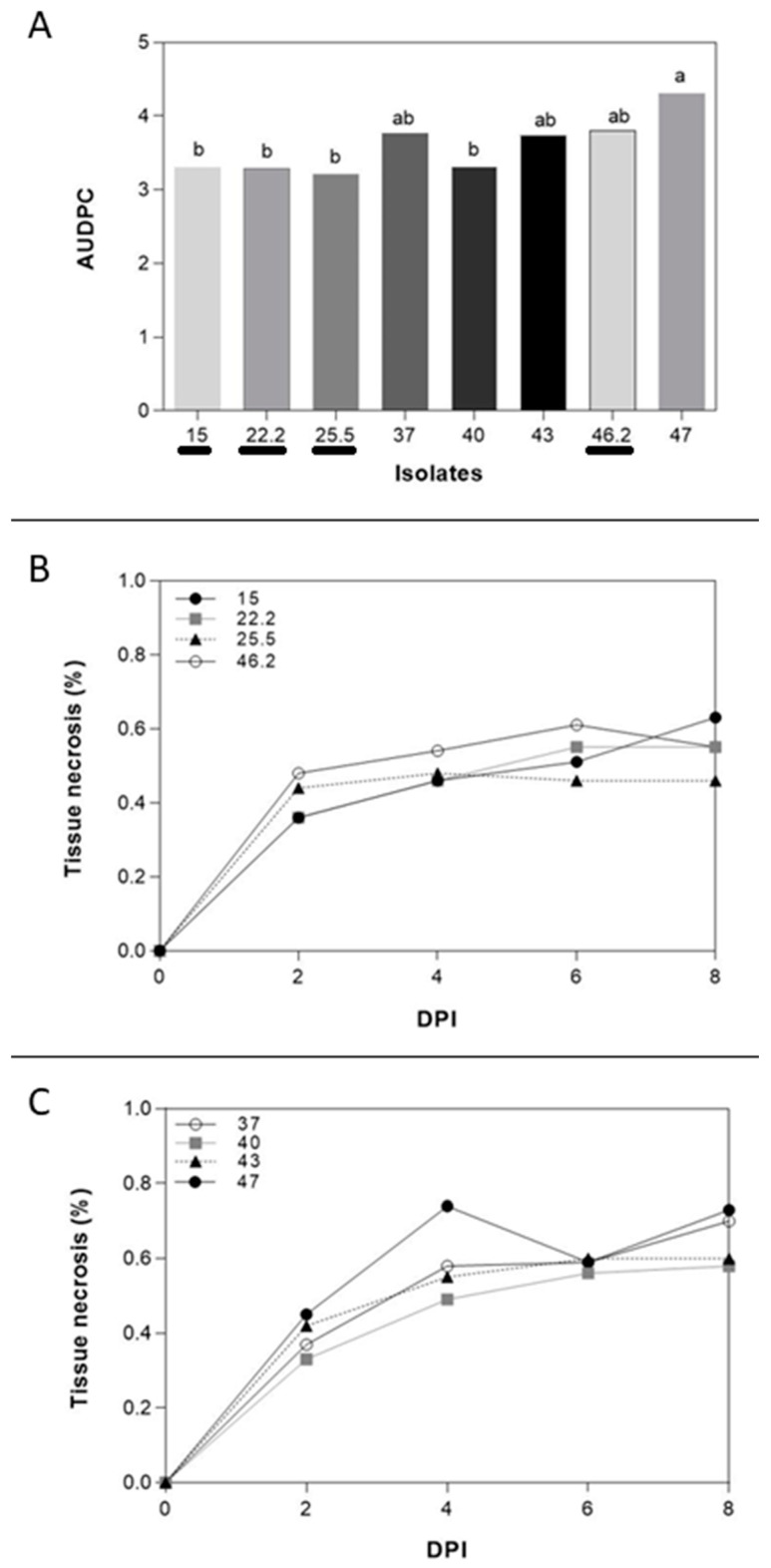
(**A**) Different AUDPC of several *F. poae* isolates from barley and wheat. Isolates from barley are underlined. (**B**,**C**) Tissue necrosis (%) of *F. poae* isolates at 2, 4, 6, and 8 dpi isolates from barley and wheat, respectively. Different letters are statistically significant according to Tukey’s test at *p* < 0.05.

The selection of *F. poae* isolates was made based on *Brachypodium* root aggressiveness. The results showed different percentages of necrosis root area of all isolates on the average of the two Bd-21 and Pakistan *Brachypodium* spp. accessions (Figure 2). The isolate N° 47 from wheat was selected due to the higher aggressiveness in *Brachypodium* spp. root (4.30% ± 1.25%). The less aggressive isolate was N° 25.5 from barley (3.22% ± 1.23%). Although the differences among Bd-21 and Pakistan accessions were not statistically significant, Bd-21 (3.52% ± 1.67%) showed less necrotic root area caused by *F. poae* than the Pakistan accession (3.66% ± 1.58%). Interestingly, although the differences were not statistically significant, the isolates from wheat (3.78% ± 1.65%) tended to be more aggressive than those obtained from barley (3.4% ± 1.51%).

### 3.2. Conidial Quantification and Disease Severity

**Figure 3:**
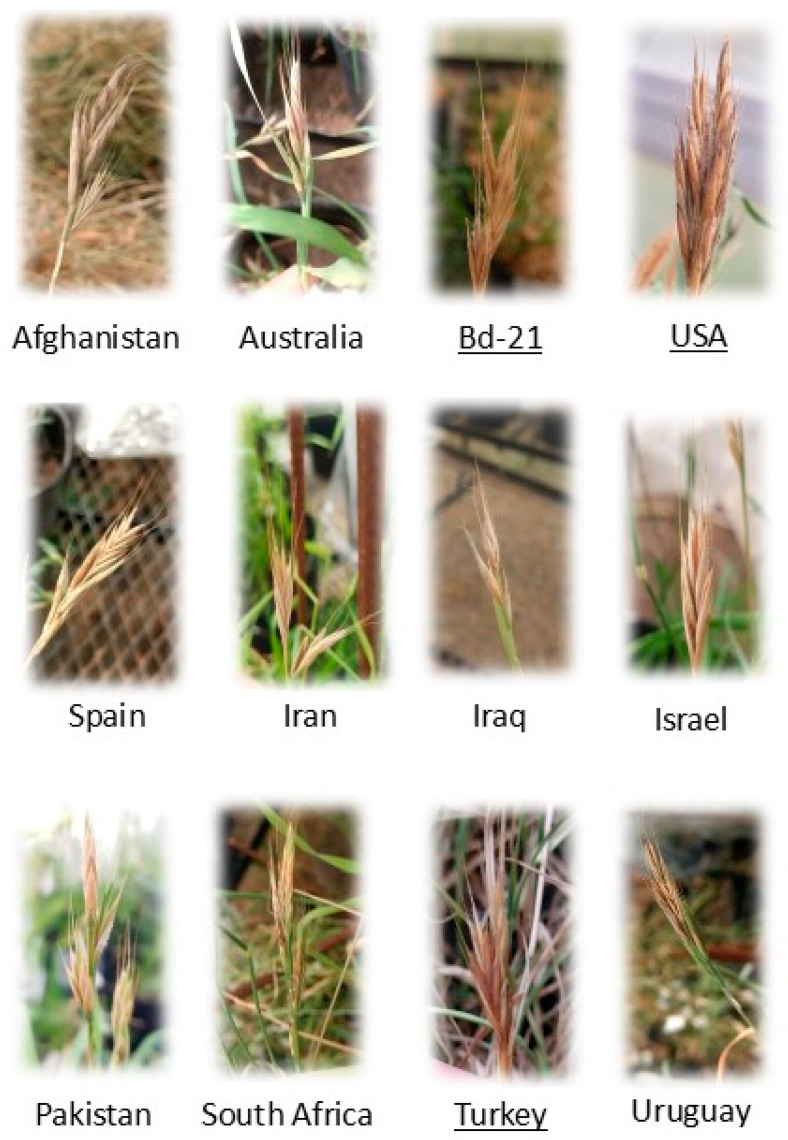
Symptoms of *F. poae* developed on *Brachypodium* accessions. The diploid accessions are underlined.

**Figure 4:**
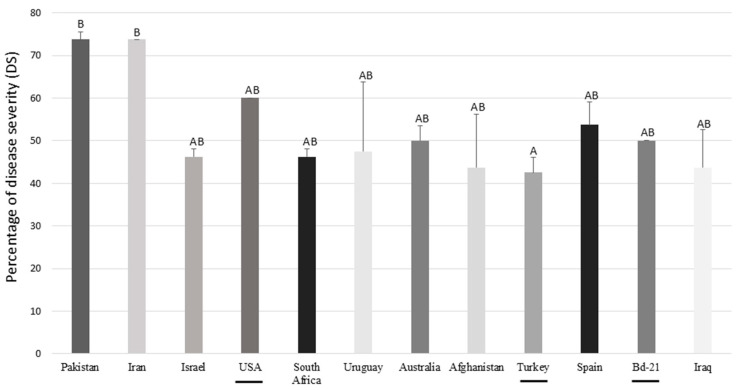
Percentage of disease severity (DS) of *Brachypodium* accessions inoculated with *F. poae*. The diploid accessions are underlined. Different letters are statistically significant according to Tukey’s test at *p* < 0.01.

As regards DS, *F. poae* developed symptoms in all *Brachypodium* accessions evaluated (Figure 3). The accessions showed different behavior against *F. poae*, showing the accession from Turkey had the lowest DS values (42.5% ± 3.54%). On the other hand, the *B. hybridum* from Pakistan and Iran were the most affected lines (73.75% ± 1.77% and 73.75% ± 8.84%, respectively) (Figure 4). Regarding the quantification of conidia, all suspensions from infected spikelets of *Brachypodium* spp. accessions presented conidia of *F. poae* regardless of the day collected. However, statistical differences were observed in the days evaluated. At 7 dpi, the number of conidia was 4.83 ± 3.71 conidia/mL, at 14 dpi, it was 13.18 ± 7.13 conidia/mL, and finally, at 21 dpi, it was 18.58 ± 12.71 conidia/mL.

### 3.3. Fusarium poae DNA and NIV Quantification

The results obtained from the analysis showed a significant difference between the *Brachypodium* accessions. Two groups were separated based on statistical differences: the Pakistan and the remaining *Brachypodium* accessions. The Pakistan accession showed the most significant amount of *F. poae* DNA with a mean score of 2.82 ng ± 0.76 ng *F. poae* DNA/10 ng total DNA. Among the other group, Turkey was the accession with more *F. poae* DNA content (0.012 ng ± 0.01 ng), followed by Afghanistan (0.010 ng ± 0.004 ng). The accessions of Australia (0.009 ng ± 0.008 ng), Iran (0.008 ng ± 0.002 ng), Iraq (0.007 ng ± 0.001 ng), Spain (0.006 ng ± 0.0005 ng), South Africa (0.006 ng ± 0.003 ng), Uruguay (0.003 ng ± 0.001 ng), Bd-21 (0.001 ng ± 0.002 ng), and Israel (0.001 ng ± 0.001 ng) showed intermediate values of DNA content. Finally, USA accessions did not show *F. poae* DNA content. The analysis of mycotoxins found that all the *Brachypodium* accessions showed no detectable NIV quantities (>10 ng/g).

## 4. Discussion

In simple practice, *Brachypodium* spp. has been recognized as an emerging system model. It is a small plant that is easy to maintain, but can develop several important cereal diseases, resulting in useful aspects for research [54]. Conversely, *F. poae* has been isolated with a high frequency in cereal grains worldwide [23,26,55,56]. Likewise, another study revealed that *F. poae* was the major fungal from wheat samples originating from Poland [57]. Currently, no information is available on the impact of *F. poae* on the *Brachypodium* spp. Therefore, our work aimed to determine the interaction of *Brachypodium*-*F. poae* to be useful for future plant–pathogen interaction studies.

For these studies, *F. poae* isolates from different crops (barley and wheat) were used. Our results demonstrate that the *F. poae* isolates were aggressive against Bd-21 and Pakistan *Brachypodium* roots. However, the isolates obtained from wheat showed more aggressiveness compared with those obtained from barley. Moreover, the isolate N°47 from wheat presented significant differences in the necrosis of the roots of the two accessions of *Brachypodium*, being selected for pathogenicity assay. Several works have shown that *F. poae* is an FHB pathogen affecting both crops, although barley is less frequently affected than wheat [22,26]. Differences in temporal and spatial flowering patterns among crops could be responsible for the differences observed [23]. In wheat, conidia of *Fusarium* spp. are deposited on or inside wheat spikes, germinating and initiating infection [58,59]. However, in barley, the fungus spreads from the exterior of the spikes under wet conditions, and internal spread in the rachis is more limited [60].

Regarding *Brachypodium*–*Fusarium poae* pathogenicity assays, the inoculation in floral tissues was used as a classical method for evaluation of the aggressiveness of *Fusarium* spp. [61]. In our study, this technique allowed the development of symptoms in all the accessions inoculated with *F. poae*. Moreover, the conidial quantification and fungal DNA biomass showed different results among accessions, which could indicate that some accessions respond differently to the pathogen presence. It would be interesting to evaluate the expression of defense genes among accessions inoculated with the pathogen to know the differences in responses observed. Compatible interaction among *Brachypodium* and different fungal pathogenic species has been confirmed. For example, the potential use of *Brachypodium* as a plant model for discovering genetic variation in resistance to *Rhizoctonia solani* was demonstrated [62]. Also, *Brachypodium* was used as a model plant against *Puccinia graminis* [63]. Moreover, various reported studies showed compatible interactions between *Brachypodium* and *Claviceps purpurea*, *Ramularia collo cygni*, *Oculimacula* spp., *Magnaporthe grisea*, *Cochliobolus sativus*, *Gaeumannomyces graminis*, *Pyrenophora teres*, *Stagonospora nodorum*, and *Colletotrichum cereale* [34,64,65].

Regarding *Fusarium* species, *F. graminearum*, *F. cerealis*, *F. pseudograminearum*, and *F. poae* showed interaction with *Brachypodium* roots [3]. Similarly, the interaction between *Brachypodium* and the most prevalent species of *Fusarium* in Europe, *F. graminearum*, and *F. culmorum*, has been evaluated [15]. These results showed not only the capacity of these species to develop symptoms on root, coleoptile, and foliar tissues but also that the plant model exhibited characteristics of susceptibility similar to those of wheat. Likewise, transcriptomic and metabolomic assays to evaluate the interaction between *Brachypodium* and two isolates of *F. graminearum*, a wild-type producing deoxynivalenol (DON) mycotoxin and another impaired in DON production, was developed [14]. The results showed an extensive colonization of the pathogen over *Brachypodium*. Moreover, researchers demonstrated the capacity of DON to act as a virulence factor, as the isolate producing the mycotoxin was more aggressive than the isolate impaired in the production of DON. *Fusarium poae* cannot produce DON, but this species can produce NIV. In our study, the samples evaluated did not have detectable NIV quantities. The temperature range favoring the production of NIV by *F. poae* was between 25 °C and 35 °C (the estimated optimum being 27.5 °C), which is different from the growth temperature of the fungus [66]. The mean minimum and maximum temperatures registered in our assay were 15.46 °C and 34.80 °C, respectively. Although the range of temperatures supports NIV production by *F. poae*, not only this variable favors the production of secondary metabolites called mycotoxin, but also other factors such as pH, humidity, light, and interaction with other microorganisms [67]. These factors could explain the absence of NIV in the samples evaluated despite the development of the fungus. Moreover, its role as a virulence factor has been discussed, assessing the effect of trichothecenes in the virulence of the pathogen [68]. For this objective, the gene coding for the initial enzyme of trichothecenes biosynthesis (Tri5 trichodiene synthase) was disrupted in three *F. graminearum* isolates with different mycotoxin production profiles. These isolates were used as inoculums to pathogenicity assays in wheat, barley, and maize. The results showed that the role of the trichothecenes varied depending on the crop evaluated. In wheat, the Tri5 disruption significantly reduced the *F. graminearum* virulence, while in barley, the decrease was not statistically significant. Interestingly, NIV is a virulence factor in maize, where the absence of NIV production reduces disease severity. In *Brachypodium*, as described before, DON acts as a virulence factor, but the role of NIV in this crop has not been described yet [14].

## 5. Conclusions

Our results demonstrated that the model plant proposed, *Brachypodium* spp., could be useful for plant—*F. poae* interactions. Moreover, *Brachypodium* accessions responded differently against *F. poae*. It would be interesting for future work to elucidate whether the *Brachypodium* responses correspond to some defense genes that activate in pathogen presence in some accessions, which could explain better the differential behavior against *F. poae*.

## Data Availability

The original contributions presented in the study are included in the article, further inquiries can be directed to the corresponding authors.
